# Swiss National Registry on Catheter Ablation Procedures: Changing Trends over the Last 20 Years

**DOI:** 10.3390/jcm10143021

**Published:** 2021-07-07

**Authors:** Nadine Molitor, Emre Yalcinkaya, Angelo Auricchio, Haran Burri, Etienne Delacretaz, Michael Kühne, Andrea Menafoglio, Sven Reek, Tobias Reichlin, Claudia Herrera-Siklody, Marianne Zimmerli, Christian Sticherling, Firat Duru

**Affiliations:** 1 Department of Cardiology, University Heart Center Zurich, 8091 Zurich, Switzerland; nadine.molitor@usz.ch (N.M.); dremreyalcinkaya@gmail.com (E.Y.); 2 Department of Cardiology, Istituto Cardiocentro Ticino, 6900 Lugano, Switzerland; angelo.auricchio@eoc.ch; 3 Department of Cardiology, University Hospital Geneva, 1205 Geneva, Switzerland; Haran.Burri@hcuge.ch; 4 Department of Cardiology, Hirslanden Clinique Cecil, 1011 Lausanne, Switzerland; etienne.delacretaz@svmed.ch; 5 Department of Cardiology, University Hospital Basel, 4031 Basel, Switzerland; Michael.Kuehne@usb.ch (M.K.); Christian.Sticherling@usb.ch (C.S.); 6 Department of Cardiology, Hospital of Bellinzona and Valli, 6500 Bellinzona, Switzerland; andrea.menafoglio@eoc.ch; 7 Department of Cardiology, Hirslanden Clinica Aarau, 5001 Aarau, Switzerland; Sven.Reek@hirslanden.ch; 8 Department of Cardiology, Inselspital University Hospital Bern, University of Bern, 3010 Bern, Switzerland; tobias.reichlin@insel.ch; 9 Department of Cardiology, University Hospital Lausanne, 1011 Lausanne, Switzerland; Claudia.Herrera-Siklody@chuv.ch; 10 Department of Cardiology, Hospital Thun, 3600 Thun, Switzerland; Marianne.ZimmerliVoegtli@spitalstsag.ch; 11 Center for Integrative Human Physiology, University of Zurich, 8091 Zurich, Switzerland

**Keywords:** catheter ablation, electrophysiology, national registry, clinical outcome, quality assurance

## Abstract

The Swiss Ablation Registry provides a national database for electrophysiologic studies and catheter ablations. We analyzed the database to provide an in-depth look at changing trends over the last 20 years. During the study period a total of 78622 catheter ablations (age 61.0 ± 1.2 years; 63.7% male) were performed in 29 centers. The number of ablations increased by approximately ten-fold in 20 years. Ablation for atrial fibrillation (AF) was the main driver behind this increase, with more than hundred-fold (39.7% of all ablations in 2019). Atrioventricular-nodal-reentrant-tachycardia (AVNRT) and accessory pathways, being the main indications for ablation in 2000 (44.1%/25.1%, respectively), made up of only a small proportion (15.2%/3.5%,) respectively in 2019. Fluoroscopy, ablation, and procedure durations were reduced for all ablations over time. The highest repeat ablations were performed for ventricular tachycardia and AF (24.4%/24.3%). The majority of ablations (63.0%) are currently performed in private hospitals and non-university public hospitals whereas university hospitals had dominated (82.4%) at the turn of the century. A pronounced increase in the number of catheter ablations in Switzerland was accompanied by a marked decrease in fluoroscopy, ablation, and procedure durations. We observed a shift toward more complex procedures in older patients with comorbidities.

## 1. Introduction

The Swiss National Pacemaker and Ablation Registry was established in 1992 by the Pacemaker Foundation of Switzerland in order to provide a nationwide database for quality assurance. Initially, the Swiss Registry collected data in paper form for each catheter ablation in the country. In later years, the scope of the Swiss Registry was extended to include more extensive clinical data, and since 2013, the data have been collected electronically using a dedicated web platform (CH-PACE Web).

The Swiss Ablation Registry provides comprehensive information on catheter ablations, focusing on indications, patient-, and procedural characteristics. All Swiss centers performing catheter ablations are obliged to provide their data in this registry. The purpose of this study is to report the catheter ablation statistics in Switzerland and provide an in-depth look at changing trends over the course of the last 20 years.

## 2. Materials and Methods

Anonymized data were collected prospectively between 1 January 2000 and 31 December 2019. The data were collected on paper documents initially, and since 2013 these are obtained on a dedicated, secure web-based platform. The central program of the web-based platform is accessed via an Internet browser. The data are recorded online in the implanting centers after the intervention and transmitted to the central server by protected connection. On the central server, the sensitive patient data are stored in encrypted form. All Swiss centers performing catheter ablations were obliged to provide their data in this web-based platform, which made it possible to collect significantly more variables. Information on ablation indications, patient characteristics like age, sex, symptoms, comorbidities antiarrhythmic drugs and anticoagulation, procedural characteristics as well as acute success and complications were collected by this platform. The registry strictly adhered to ethical guidelines of Helsinki declaration and was approved by the responsible ethics committee without any restrictions. The integrity of the database was periodically evaluated by the Pacing and Electrophysiology Working Group of the Swiss Society of Cardiology. The data are presented using descriptive statistics with mean and standard deviation values (for continuous variables) or percentage values (for categorial variables).

## 3. Results

**Figure 1:**
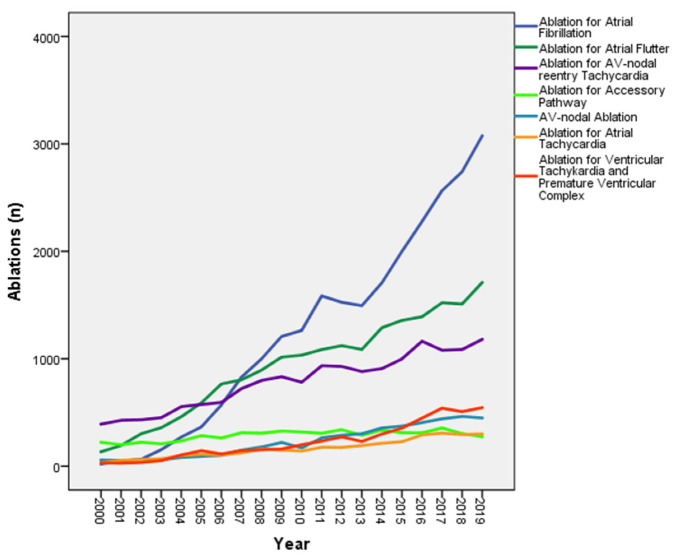
Number of ablations distributed by type of ablation from 2000 till the end of 2019.

During the study period, a total of 78,622 catheter ablations were performed (age 61.0 ± 1.2 years; 63.7% male) in 29 centers in Switzerland. The absolute number of ablations increased by approximately ten-fold in the last 20 years (Figure 1). Corrected for population density, the number of ablations increased from 12 ablations per 100,000 inhabitants to 90.4 ablations per 100,000 inhabitants (Table 1). Ablation for atrial fibrillation (AF) was the main driver behind this increase, with an increase of more than hundred-fold. 

### 3.1. Ablation Indications

Overall, the main indication for catheter ablation was AF (31.5%), followed by atrial flutter (23.8%) and AV-nodal reentrant tachycardia (AVNRT) (20.0%). Throughout the study period, 24759 AF ablation procedures were performed, mostly due to paroxysmal AF (68.3%), followed by persistent AF (29.5%) and long-standing persistent AF (2.2%). In 2019, AF ablations constituted 39.7% of all catheter ablations in Switzerland. The number of ablation procedures for atrial flutter (AFL) was 18,616 throughout the study period. These ablations were performed within the right atrium in 91% of the procedures, with typical atrial flutter involving the cavo-tricuspid isthmus being the most common arrhythmia (96.3%). Scar-related right-sided atrial flutters made up only a small proportion of the procedures (2.3%). Left sided atrial flutters most commonly involved a peri-mitral circuit (38.1%). The majority of AVNRT ablations were performed for the typical (slow-fast type) AVNRT (93.2%), whereas atypical AVNRT (slow-slow and fast-slow types) constituted 3.5 and 3.3% of the cases, respectively.

The predominant types of ablations performed in Switzerland changed over time. AVNRT and accessory pathways (AP), being the main indications for ablation in the year 2000 (44.1% and 25.1%, respectively), made up only a small proportion of ablations (15.2% and 3.5%, respectively) in 2019. The likelihood of a repeat ablation for the same arrhythmia as the index procedure, varied depending on the ablation type. The lowest rates for repeat ablation were seen in patients undergoing AV-nodal ablation and ablations for AVNRT, whereas repeat interventions were performed in more than 24% of AF cases (Table 2). 

### 3.2. Concomitant Heart Disease

The Swiss Ablation Registry provided information about the concomitant heart diseases, including hypertensive, ischemic, dilated, hypertrophic, valvular, infiltrative, congenital, and arrhythmogenic cardiomyopathies as well as various channelopathies. The rate of reported concomitant heart diseases was highest for patients undergoing ventricular tachycardia (VT) and AV-nodal ablations. For VT ablations, the most commonly reported concomitant heart disease was ischemic cardiomyopathy (44.5%), followed by dilated cardiomyopathy (10.3%), and this observation did not change over time. In contrast, in patients undergoing AF ablation, the rate of reported concomitant heart diseases increased from 30% in 2013 to 41% in 2019 (Supplementary Figure S1).

### 3.3. Age and Gender Distribution

Age and gender distributions from 2013 till the end of 2019 by type of catheter ablation are presented in Table 2. During this period, there was a consistent increase in the mean age of patients for all catheter ablation procedures. AF ablations were commonly performed in men (69.3%). A male predominance was also seen in atrial flutter (78.6%) and VTs (78.5%), followed by APs (62%) and ablations for premature ventricular contraction (PVC) (58%), whereas women more commonly underwent ablations for AVNRT (55.6%), AT (53.3%), and AV-nodal ablation (51.8%). For all ablation types, the proportion of men and women remained stable over the years. Patients undergoing catheter ablation for APs were the youngest, whereas those undergoing AV-nodal ablation were substantially older (Supplementary Figure S2). At the time of the procedure, men were significantly older than women during ablations for VT (63.6 ± 1.5 years vs. 55.4 ± 3.0 years), PVC (58.0 ± 1.5 years vs. 51.0 ± 2.4 years), and AVNRT (58.0 ± 1.6 years vs. 54.0 ± 1.1 years), while they were significantly younger during ablations for AF (61.0 ± 0.8 years vs. 65.7 ± 1.1 years) and APs (35.7 ± 1.3 years vs. 39.6 ± 1.5 years).

### 3.4. Ablation Energy

The vast majority of the catheter ablations were performed using radiofrequency (RF) energy. Ablations using cryo-energy were mostly performed for AF (11% of AF ablations) (Table 2). Absolute numbers using cryo-balloon showed a marked increase by more than twenty-fold from 20 in 2013 to 537 in 2019. In other types of ablation, cryo-energy was used for occasional cases (1.3% in AP, 0.3% in AVNRT, 0.2% in atrial flutter) during this period. 

### 3.5. Fluoroscopy, Ablation and Procedure Durations, 3D Mapping System

**Figure 2:**
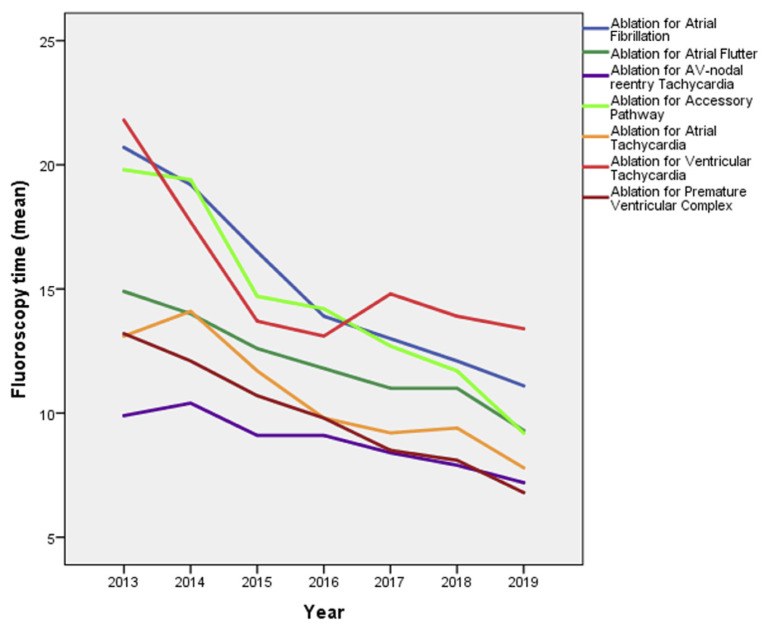
Fluoroscopy durations during from 2013 till the end of 2019.

Fluoroscopy durations were significantly reduced for all catheter ablations over the years (Figure 2). As compared to fluoroscopy duration in 2013, ablations for AF and VT required approximately 50% shorter fluoroscopy times in 2019. Overall, the shortest fluoroscopy durations were achieved for ablation of the AV node, ablation for AVNRT, PVC, and AT.

Ablation durations differed markedly depending on the complexity of the procedure, the longest being for AF (mean 30.9 min). Both point-by-point RF and cryo-balloon approaches for AF showed a significant decline in ablation duration over time. Since 2014, the shortest ablation durations were obtained by using cryo-energy. VT ablations required a mean duration of 21.5 min.

Procedure durations also decreased markedly over the years. While atrial flutter and AVNRT ablations required the shortest procedure durations (mean 72.6 min and 73.8 min, respectively), VT ablations required the longest time (mean 171.17 min), which remained more or less stable over time. 

Over the years, we have observed an increasing trend for the use of 3D-mapping systems. In 2013, 3D-mapping systems were commonly used for the majority of complex procedures such as AF or VT ablation, but in procedures like AVNRT or right atrial flutter ablation, these systems were rarely used (0.6% and 8.1%, respectively). In 2019, 3D mapping systems were used in more than 42% of all AVNRT and right atrial flutter ablations, further explaining the reason of the reduction of fluoroscopy exposures in recent years.

### 3.6. Acute Procedural Success Rates

The ablation procedure was considered successful when the electrophysiological end-point of the ablation procedure was reached. For example, successful electrical isolation of all pulmonary veins was considered acute success for AF. Likewise, for atrial flutter, successful block of the flutter isthmus was considered acute success. For AVNRT and other arrhythmias, acute success was defined as the non-inducibility of the clinical arrhythmia at the end of the procedure. The data were available as provided by the ablating center and were not confirmed by external auditing. 

The highest reported acute procedural success rates were reported for AVNRT (99.0%), AF (99.0%), and atrial flutter (96.8%) in the 2013–2019 period (Supplementary Figure S3). The acute success rates remained stable (AP, AVNRT, AF) or showed an increase over the years (AFL, AT, PVC, VT). VT ablation showed the strongest increase (42% to 55%) in the 2013–2019 period.

### 3.7. Location and Type of Ablation Centers

**Figure 3:**
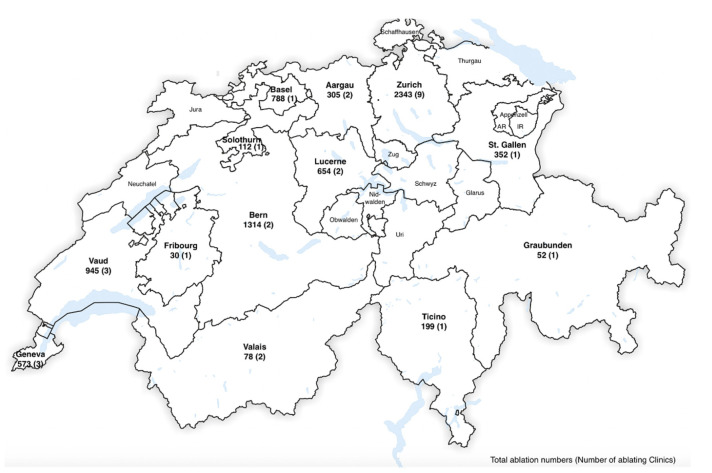
Number of catheter ablations and ablating centers across Swiss cantons (2019 data).

The locations of ablation centers in Switzerland and the number of catheter ablations across various cantons are shown in Figure 3. The highest number of ablations were performed in Canton Zurich (2343), followed by Cantons Bern (1314), Vaud (945), Basel (788), and Lucerne (654) (2019 data). 

**Figure 4:**
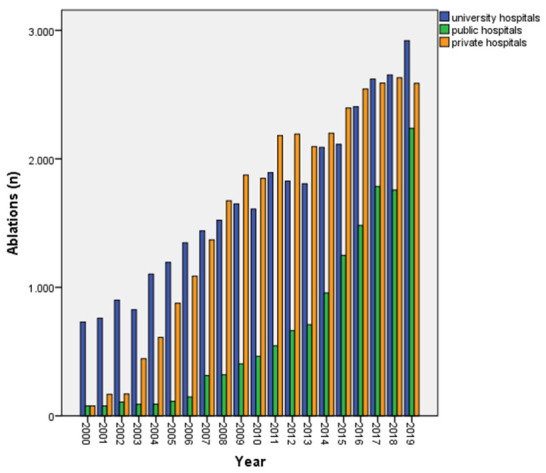
Ablations distributed to ablating centers from 2000 till the end of 2019.

Of the participating centers, 42.5% were university hospitals, 40.2% were private hospitals, and 17.3% were non-university public hospitals. By the end of the study period, 63.0% of all ablations in Switzerland were performed in private or public hospitals. This was in sharp contrast to earlier years, when university hospitals had dominated the scene (82.4%) at the turn of the century (Figure 4).

In the final study year, most of ablating centers (86.2%) were performing AF ablations. VT ablations were performed by 68.9% of all clinics. However, only 35% of all ablating centers performed more than 10 VT ablations per year. Among all VT ablations, 66.3% were performed in university hospitals.

## 4. Discussion

This is the first comprehensive report on catheter ablation statistics in Switzerland based on the data in the Swiss National Pacemaker and Ablation Registry. This registry was unique because it was consistently utilized throughout the last 20 years by all implanting centers in the country. Moreover, there was a significant upgrade with the use of secure, web-based platform for data entry in the last seven years. The study showed a continuous increase in ablation procedures and a marked decrease in fluoroscopy, ablation and procedure durations in the country throughout the study period. Ablation for AF was the main driver behind this increase and constituted the most common indication for catheter ablations in the country in recent years.

Our findings regarding the annual number of ablation procedures are in accordance with the temporal trends observed in previously published reports. A publication from the Swedish National Registry showed a 138% increase (from 1953 to 4648) in the yearly ablation volume over a 10-year period (2006 until 2015). Although the numbers increased for all ablation procedures, the major increase was due to AF procedures (430% increase). In the last year of the study, 40% of all ablations were performed for AF [1]. A similar trend (171% increase in the 2005–2014 period) was reported in the Danish Ablation Registry [2]. They reported a 171% increase in the number of first-time AF ablation procedures over the study period. Likewise, in our Swiss Ablation Registry, the very significant increase in annual procedure volume over the 20-year study period was primarily due to an increase in the number of AF ablations. In the final year of our registry (2019), AF ablations accounted for 39.7% of all ablation procedures.

In addition to AF ablations, we have also observed a significant increase in the number atrial flutter ablations over the last 20 years. A possible reason for this trend maybe better detection and routine screening for AF, during which atrial flutter can also be detected. Furthermore, atrial flutter is actively looked for as part of an AF ablation and is often treated at the same time, if clinically considered necessary.

In our registry, the observed shift for more complex ablation procedures was also the case for VT ablations. This increase can be explained, at least partly, by the increasing age of the patient population along with more common comorbidities predisposing to AF [3]. This trend was also in line with the reported better success rates in recent years along with broader recommendations for catheter ablation in the recent guidelines. While catheter ablation cures most supraventricular tachycardias, ablation for AF can provide better symptom control and an improvement in quality-of-life, and hence, has been more commonly recommended by physicians in recent years [4,5,6]. Since a recent multicenter trial has shown that early rhythm control strategy, as compared to usual care, which is predominantly based on rate control, is associated with a lower risk of adverse cardiovascular outcomes, the number of future ablations for AF is likely to further increase in the coming years [7].

The reported acute procedural success rates for AVNRT, AF, and isthmus-dependent atrial flutter in our registry varied from 96% to 99%. These rates were 92% and 86% for ablations for AP and AT ablations, respectively. Brachmann et al. reported similar acute success rates for supraventricular ablations in the German Ablation Registry on 12566 patients enrolled between 2007 and 2010 [8]. In this study, the reported success rates were over 94% for all supraventricular ablations except for AT (84.3%). The reported success rate for PVC ablations (85%) in the Swiss Registry also matched the results reported by Latchamsetty at al. in a multicenter retrospective cohort study (1185 patients, 84% acute success) [9].

In our registry, 48% of patients undergoing VT ablation were reported to be non-inducible at the end of the procedure, with an increase from 42% to 55% in the 2013–2019 period. The endpoints for VT ablation were not defined uniformly in various studies; nevertheless, non-inducibility of clinical VT and any VT were both considered to be the best outcome predictors [10]. In a meta-analysis, non-inducibility of any VT was shown to be associated with improved arrhythmia-free survival and all-cause mortality [11]. The reported non-inducibility rates for any VT ranges between 29 and 83% in the literature, [11,12] with variable study sizes, large diversity of patient and substrate characteristics, as well as ablation strategies, limiting the possibility for direct comparisons even in prospective randomized studies. Three large, prospective, multicenter trials reported acute success rates (defined as elimination of all inducible VTs) of 41%, 49%, and 60% [13,14,15]. The results for acute VT ablation success rates in the Swiss Ablation Registry are comparable with the above-mentioned reports.

The high acute success rate for AF ablation in our registry refers to technical success of the procedure with successful isolation of all pulmonary veins, but it certainly does not reflect to clinical success in the short or long term. AF ablation procedures were performed, mostly due to paroxysmal AF (68.3%), followed by persistent AF (29.5%) and long-standing persistent AF (2.2%), in accordance with the numbers reported from the Spanish Ablation Registry [16]. Despite constant improvements in acute success rates, the number of redo interventions for AF ablations remained stable over the years in the country, which were possibly due to the increasing age of patients and the number of concomitant heart diseases. We observed an increase in the mean age of patients undergoing catheter ablation procedures over the years, reflecting aging of the population due to increase in life expectancy and extended indications for catheter ablation, especially for symptomatic AF, which has been strongly recommended in the recent European Society of Cardiology guidelines [5,6]. As expected, the rate of reported concomitant cardiac diseases increased from 30% in 2013 to 41% in 2019 in our registry. The CASTLE-AF study has shown that patients with concomitant cardiomyopathies may benefit from AF ablation but show higher recurrence rates (50% in 60 months) compared to interventions in patients with normal hearts [17].

Similar to other published studies, we observed that most patients (63.7%) undergoing catheter ablation in Switzerland were male [18,19]. The only exceptions were for AVNRT and AT ablations, which were more commonly performed in female patients, a finding consistent with the higher prevalence of these tachyarrhythmias tachycardias in women [20,21]. On the other hand, women seem to undergo overall fewer catheter ablations and be treated later in life compared to men. This might partially be due to the fact that AF is the main indication for catheter ablation and female patients are generally older when they develop AF [22]. However, females are likely disadvantaged due to other non-medical reasons. In a large registry study from the United States, female gender and belonging to a minority (Hispanic or African American) were the strongest predictors of not being ablated [23]. Other studies have also shown a disproportionately lower proportion of females undergoing AF ablation [24,25,26]. Referral bias is the most likely explanation for these observations, whereas reluctance of some women themselves toward invasive therapies may also play some role. 

Our registry showed, in consistence with other reports, significant reductions in fluoroscopy, ablation, and procedure durations over time [1]. Increasing operator experience, advances in ablation techniques and equipment, as well as the widespread use of electroanatomic mapping are among the factors that likely contribute to these developments. Only VT ablations showed a slight increase in procedure durations, possibly due to increasing numbers of more complex procedures in patients with diverse substrates.

Currently, the ablation procedures in Switzerland are distributed almost equally among university hospitals, private hospitals, and non-university public hospitals, whereas university hospitals had dominated the scene at the turn of the century. More complex interventions like VT ablations, are still most often performed in university hospitals (69%), particularly since they typically have larger teams and offer broad interdisciplinary expertise.

This report has the limitation of being based on gathered data, which are self-reported in a large nationwide registry. Therefore, we cannot exclude minor inconsistencies due to interpretation of success and other parameters among centers and operators. Nevertheless, the full availability of the data in the open web, constant efforts of the Swiss Working Group on Pacemaker and Electrophysiology on assuring data integrity and cross-checks through the industry contribute to completeness and accuracy of the provided data. Our registry does not provide data on long-term success rates since no symptomatic follow-data are captured. De novo and repeat cases were assessed in the registry for each year.

## 5. Conclusions

In the last 20 years, there was a pronounced increase in the number of catheter ablations in Switzerland. This was accompanied by a marked decrease in fluoroscopy, ablation, and procedure durations. AVNRT was the most common indication in earlier years whereas AF is currently the most commonly targeted arrhythmia. We observed a shift toward more complex procedures in older patients with comorbidities. Repeat ablations are required for VT and AF in approximately one-fourth of the cases. Overall, nearly two-thirds of the catheter ablations were performed in male patients. University hospitals, once performing the majority of the cases, have now been outnumbered by private and public hospitals.

## Figures and Tables

**Table 1:** Relative ablation numbers to the yearly population of Switzerland 2000–2019.

Year	Number of Ablations (Absolute Numbers)	Number of Ablations per 100,000 Inhabitants
2019	7745	90.37
2018	7041	82.40
2017	6996	82.46
2016	6431	76.38
2015	5758	69.15
2014	5244	63.66
2013	4612	56.66
2012	4683	58.25
2011	4620	58.08
2010	3920	49.81
2009	3929	50.46
2008	3517	45.66
2007	3123	41.13
2006	2581	34.37
2005	2185	29.29
2004	1805	24.34
2003	1362	18.50
2002	1178	16.11
2001	1006	13.86
2000	886	12.30

**Table 2:** Baseline characteristics: Catheter ablation for various arrhythmias (2013–2019 data).

Type of Ablation	Male Age Mean (SD)	Female Age Mean (SD)	Difference of Mean Age between Sexes (*p*-Value)	Male N (%)	Female N (%)	All Ablations Single and Combined Investigations (n)	Ablation Single Approach (n)	Ablation Using Cryo-Energy (n)	Redo- Intervention N (%)	Without CM N (%)	Acute Success (%)
**AF**	61 (+/− 0.8)	65.7 (+/− 1.1)	<0.001	8515 (69.3)	3769 (30.7)	15,859	12,365	1754	3007 (24.3)	8023 (64.9)	99.17
**AFL**	67 (+/− 0.8)	68.4 (+/− 1.1)	0.025	5158 (78.6)	1406 (21.4)	9863	6582	21	546 (8.3)	3147 (47.8)	96.82
**AVNRT**	58 (+/− 1.6)	54 (+/− 1.1)	0.001	3013 (44.4)	3780 (55.6)	7295	6809	19	387 (5.7)	5858 (86.0)	98.93
**AP**	35.7 (+/− 1.3)	39.6 (+/− 1.5)	0.001	1256 (62.0)	796 (38.0)	2177	2060	29	302 (14.7)	1938 (94.0)	92.51
**AT**	58 (+/− 2.6)	58 (+/− 2.3)	0.906	596 (46.7)	679 (53.3)	1825	1280	0	204 (15.9)	855 (66.8)	86.31
**AVN**	74.1 (+/− 1.5)	77.6 (+/− 0.8)	<0.001	1254 (48.2)	1350 (51.8)	2789	2617	0	144 (5.5)	683 (26.1)	-
**VT**	63.6 (+/− 1.5)	55.4 (+/− 3.0)	<0.001	1155 (78.5)	316 (21.5)	1561	1489	0	364 (24.4)	407 (27.3)	48.75
**PVC**	58 (+/− 1.5)	51 (+/− 2.4)	<0.001	740 (58.0)	535 (42.0)	1361	1286	0	159 (12.4)	781 (60.7)	85.28

## Data Availability

The anonymous data underlying this article are available in the annual ablation statistics of the Swiss Rhythmology Foundation (CH-PACE Web Registry): http://www.rhythmologie-stiftung.ch/statistiken_de.html (accessed on 3 July 2021).

## Supplementary Materials

The following are available online at https://www.mdpi.com/article/10.3390/jcm10143021/s1. Figure S1: Repeat interventions and concomitant heart diseases in patients who underwent ablation for atrial fibrillation. Figure S2. Age at ablation time distributed by type of ablation from 2013 till the end of 2019. Figure S3. Acute success rates for different indications.

## References

[1] Holmqvist F., Kesek M., Englund A., Blomström-Lundqvist C., Karlsson L.O., Kennebäck G., Poçi D., Samo-Ayou R., Sigurjónsdóttir R., Ringborn M. (2019). A decade of catheter ablation of cardiac arrhythmias in Sweden: Ablation practices and outcomes. Eur. Heart J..

[2] Pallisgaard J.L., Gislason G.H., Hansen J., Johannessen A., Torp-Pedersen C., Rasmussen P.V., Hansen M.L. (2018). Temporal trends in atrial fibrillation recurrence rates after ablation between 2005 and 2014: A nationwide Danish cohort study. Eur. Heart J..

[3] Schnabel R.B., Yin X., Gona P., Larson M.G., Beiser A.S., McManus D.D., Newton-Cheh C., Lubitz S.A., Magnani J.W., Ellinor P.T. (2015). 50 year trends in atrial fibrillation prevalence, incidence, risk factors, and mortality in the Framingham Heart Study: A cohort study. Lancet.

[4] Brugada J., Katritsis D.G., Arbelo E., Arribas F., Bax J.J., Blomstrom-Lundqvist C., Calkins H., Corrado D., Deftereos S.G., Diller G.P. (2020). 2019 ESC Guidelines for the management of patients with supraventricular tachycardiaThe Task Force for the management of patients with supraventricular tachycardia of the European Society of Cardiology (ESC). Eur. Heart J..

[5] Hindricks G., Potpara T., Dagres N., Arbelo E., Bax J.J., Blomström-Lundqvist C., Boriani G., Castella M., Dan G.A., Dilaveris P.E. (2020). 2020 ESC Guidelines for the diagnosis and management of atrial fibrillation developed in collaboration with the European Association for Cardio-Thoracic Surgery (EACTS). Eur. Heart J..

[6] Kirchhof P., Benussi S., Kotecha D., Ahlsson A., Atar D., Casadei B., Castella M., Diener H.C., Heidbuchel H., Hendriks J. (2016). 2016 ESC Guidelines for the management of atrial fibrillation developed in collaboration with EACTS. Eur. Heart J..

[7] Kirchhof P., Camm A.J., Goette A., Brandes A., Eckardt L., Elvan A., Fetsch T., Van Gelder I.C., Haase D., Haegeli L.M. (2020). Early Rhythm-Control Therapy in Patients with Atrial Fibrillation. N. Engl. J. Med..

[8] Brachmann J., Lewalter T., Kuck K.H., Andresen D., Willems S., Spitzer S.G., Straube F., Schumacher B., Eckardt L., Danilovic D. (2017). Long-termsymptom improvement and patient satisfaction following catheter ablation of supraventricular tachycardia: Insights from the German ablation registry. Eur. Heart J..

[9] Latchamsetty R., Yokokawa M., Morady F., Kim H.M., Mathew S., Tilz R., Kuck K.-H., Nagashima K., Tedrow U., Stevenson W.G. (2015). Multicenter outcomes for catheter ablation of idiopathic premature ventricular complexes. JACC Clin. Electrophysiol..

[10] Santangeli P., Frankel D.S., Marchlinski F.E. (2014). Advances in Arrhythmia and Electrophysiology End Points for Ablation of Scar-Related Ventricular Tachycardia. Circulation.

[11] Ghanbari H., Baser K., Yokokawa M., Stevenson W., Della Bella P., Vergara P., Deneke T., Kuck K.H., Kottkamp H., Fei S. (2014). Noninducibility in Postinfarction Ventricular Tachycardia as an End Point for Ventricular Tachycardia Ablation and Its Effects on Outcomes. Circulation.

[12] Tanner H., Hindricks G., Volkmer M., Furniss S., Kühlkamp V., Lacroix D., De Chillou C., Almendral J., Caponi D., Kuck K.-H. (2009). Catheter Ablation of Recurrent Scar-Related Ventricular Tachycardia Using Electroanatomical Mapping and Irrigated Ablation Technology: Results of the Prospective Multicenter Euro-VT-Study. J. Cardiovasc. Electrophysiol..

[13] Calkins H., Epstein A., Packer D., Arria A., Hummel J., Gilligan D.M., Trusso J., Carlson M., Luceri R., Kopelman H. (2000). Catheter Ablation of Ventricular Tachycardia in Patients With Structural Heart Disease Using Cooled Radiofrequency Energy Results of a Prospective Multicenter Study. J. Am. Coll. Cardiol..

[14] Stevenson W.G., Wilber D.J., Natale A., Jackman W.M., Marchlinski F.E., Talbert T., Gonzalez M.D., Worley S.J., Daoud E.G., Hwang C. (2008). Irrigated Radiofrequency Catheter Ablation Guided by Electroanatomic Mapping for Recurrent Ventricular. Circulation.

[15] Kuck K.H., Schaumann A., Eckardt L., Willems S., Ventura R., Delacrétaz E., Pitschner H.F., Kautzner J., Schumacher B., Hansen P.S. (2010). Catheter ablation of stable ventricular tachycardia before defi brillator implantation in patients with coronary heart disease (VTACH): A multicentre randomised controlled trial. Lancet.

[16] Quesada A., Cózar R., Anguera I. (2020). Spanish Catheter Ablation Registry. 19th Official Report of the Heart Rhythm Association of the Spanish Society of Cardiology (2019). Rev. Española Cardiol..

[17] Marrouche N.F., Brachmann J., Andresen D., Siebels J., Boersma L., Jordaens L., Merkely B., Pokushalov E., Sanders P., Proff J. (2018). Catheter ablation for atrial fibrillation with heart failure. N. Engl. J. Med..

[18] Curley M., Berger M., Roth J., Benjamin I., Rubenstein J. (2014). Predictors of Mortality and Major In-Hospital Adverse Events Associated With Electrophysiology Catheter Ablation. JAMA Intern. Med..

[19] Bohnen M., Stevenson W.G., Tedrow U.B., Michaud G.F., John R.M., Epstein L.M., Albert C.M., Koplan B.A. (2011). Incidence and predictors of major complications from contemporary catheter ablation to treat cardiac arrhythmias. Heart Rhythm..

[20] Suenari K., Hu Y.F., Tsao H.M., Tai C.T., Chiang C.E., Lin Y.J., Chang S.L., Lo L.W., Ta-Chuan T.U.A.N., Lee P.C. (2010). Gender Differences in the Clinical Characteristics and Atrioventricular Nodal Conduction Properties in Patients With Atrioventricular Nodal Reentrant Tachycardia. J. Cardiovasc. Electrophysiol..

[21] Porter M.J., Morton J.B., Denman R., Lin A.C., Tierney S., Santucci P.A., Cai J.J., Madsen N., Wilber D.J. (2004). Influence of age and gender on the mechanism of supraventricular tachycardia. Heart Rhythm..

[22] Go A.S., Hylek E.M., Phillips K.A., Chang Y., Henault L.E., Selby J.V., Singer D.E. (2001). Prevalence of Diagnosed Atrial Fibrillation in Adults. JAMA.

[23] Patel N., Deshmukh A., Thakkar B., Coffey J.O., Agnihotri K., Patel A., Ainani N., Nalluri N., Patel N., Patel N. (2016). Gender, Race, and Health Insurance Status in Patients Undergoing Catheter Ablation for Atrial Fibrillation. Am. J. Cardiol..

[24] Patel D., Mohanty P., Di Biase L., Sanchez J.E., Shaheen M.H., Burkhardt J.D., Bassouni M., Cummings J., Wang Y., Lewis W.R. (2010). Outcomes and complications of catheter ablation for atrial fibrillation in females. Heart Rhythm..

[25] Verma A., Jiang C.-Y., Betts T.R., Chen J., Deisenhofer I., Mantovan R., Macle L., Morillo C.A., Haverkamp W., Weerasooriya R. (2015). Approaches to Catheter Ablation for Persistent Atrial Fibrillation. N. Engl. J. Med..

[26] Zylla M.M., Brachmann J., Lewalter T., Hoffmann E., Kuck K.-H., Andresen D., Willems S., Eckardt L., Tebbenjohanns J., Spitzer S.G. (2016). Sex-related outcome of atrial fibrillation ablation: Insights from the German Ablation Registry. Heart Rhythm..

