# Supplementary material for: Swiss National Registry on Catheter Ablation Procedures: Changing Trends over the Last 20 Years
Source: J Clin Med. 2021 Jul 7;10(14):3021. doi: 10.3390/jcm10143021 (PMC8304673; doi:10.3390/jcm10143021)
Supplement: Supplementary file 1 [file jcm-10-03021-s001.zip › jcm-1265037-supplementary.pdf]

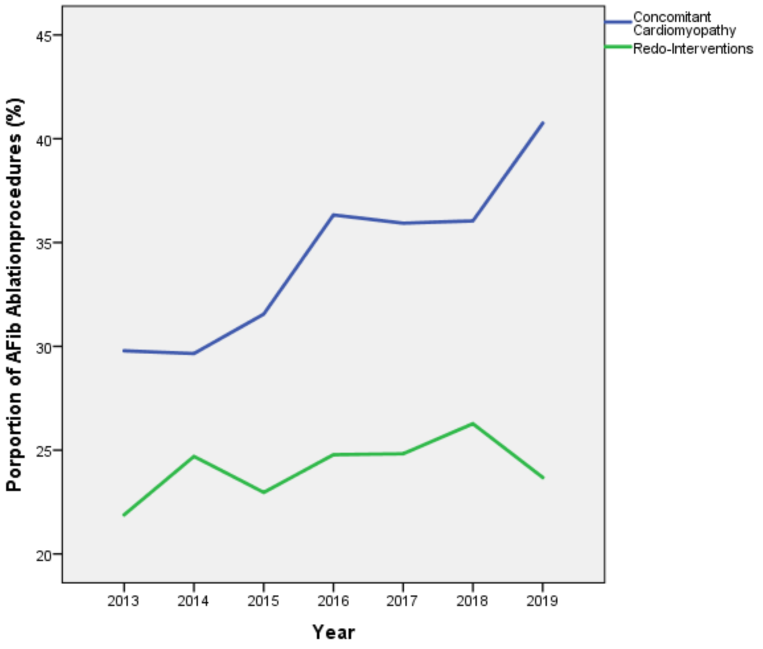


**Figure S1.** Repeat interventions and concomitant heart diseases in patients who underwent ablation for atrial fibrillation.

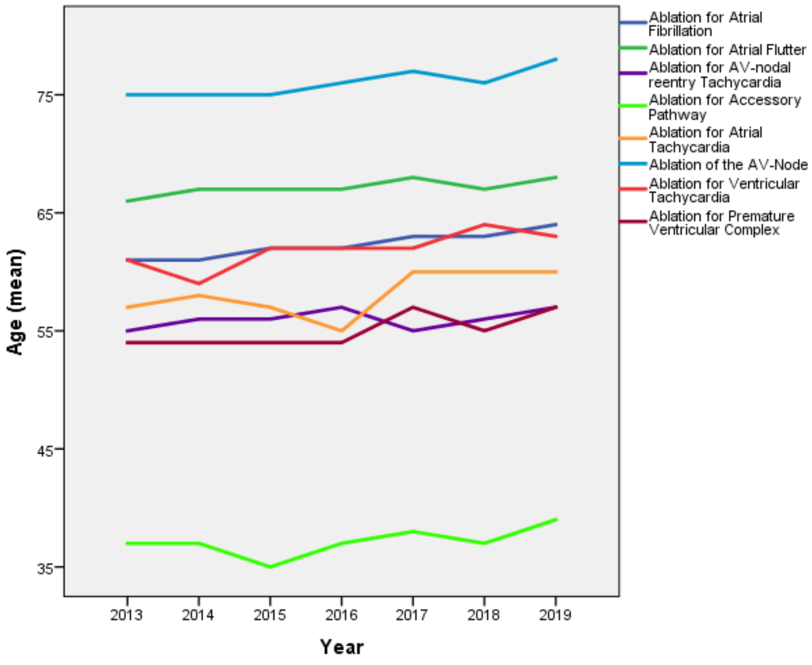


**Figure S2.** Age at ablation time distributed by type of ablation from 2013 till the end of 2019.

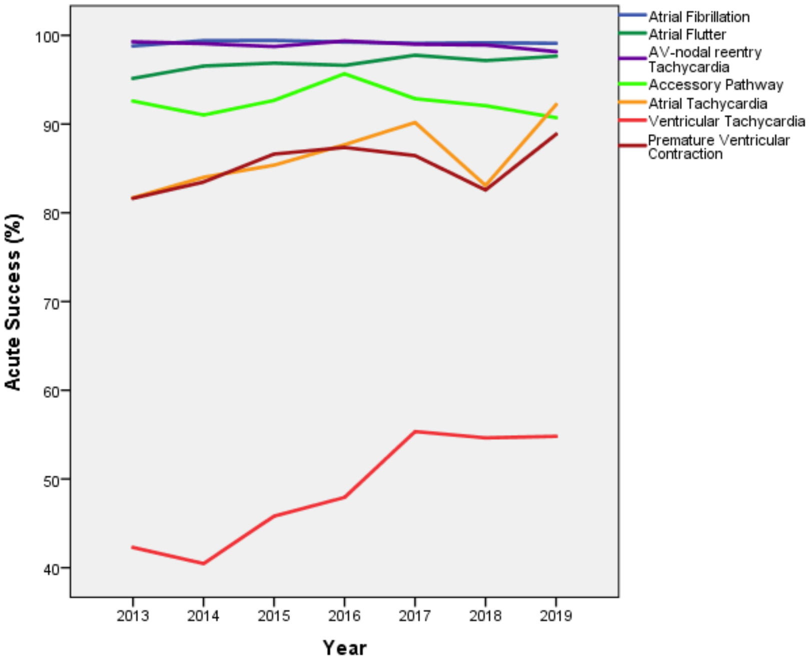


**Figure S3.** Acute success rates for different indications.
